# Repeated high blood pressure at 6 and 11 years at the Pelotas 2004 birth cohort study

**DOI:** 10.1186/s12889-019-7544-0

**Published:** 2019-09-12

**Authors:** Marília Cruz Guttier, Raquel Siqueira Barcelos, Rodrigo Wiltgen Ferreira, Caroline Cardozo Bortolotto, William Jones Dartora, Maria Inês Schmidt, Alicia Matijasevich, Luciana Tovo-Rodrigues, Iná S. Santos

**Affiliations:** 1 Postgraduate Program in Epidemiology, Federal University of Pelotas (UFPel), Cep: 96020-220 - Caixa Postal 464, Rua Marechal Deodoro, 1160 - 3° Piso. Bairro Centro, Pelotas, Rio Grande do Sul Brazil; 2 Postgraduate Program in Physical Education, Federal University of Pelotas (UFPel), Pelotas, Rio Grande do Sul Brazil; 3 Postgraduate Program in Epidemiology, Federal University of Rio Grande do Sul (UFRGS), Porto Alegre, Rio Grande do Sul Brazil; 4 Department of Preventive Medicine, Faculty of Medicine, University of São Paulo (USP), São Paulo, Brazil

**Keywords:** Blood pressure, Arterial pressure, Childhood, Cohort study

## Abstract

**Background:**

We evaluated the prevalence and the factors associated with repeated high systolic (SBP) and diastolic blood pressure (DBP) at 6- and 11-year follow-ups of children from the Pelotas (Brazil) 2004 Birth Cohort.

**Methods:**

All live births to mothers living in the urban area of Pelotas were enrolled in the cohort. Blood pressure (BP) values were transformed into Z-scores by sex, age, and height. High SBP and DBP were defined as repeated systolic and diastolic BP Z-scores on the ≥95th percentile at the two follow-ups. Prevalence (95% confidence interval) of repeated high SBP, DBP, and both (SDBP) were calculated. Associations with maternal and child characteristics were explored in crude and adjusted logistic regression analyses.

**Results:**

A total of 3182 cohort participants were analyzed. Prevalence of repeated high SBP, DBP and SDBP was 1.7% (1.2–2.1%), 2.3% (1.8–2.9%) and 1.2% (0.9–1.6%), respectively. Repeated high SBP was associated with males, gestational diabetes mellitus (2.92; 1.13–7.58) and obesity at 11 years (2.44; 1.29–4.59); while repeated high DBP was associated with females, family history of hypertension from both sides (3.95; 1.59–9.85) and gestational age < 34 weeks (4.08; 1.52–10.96). Repeated high SDBP was not associated with any of the characteristics investigated.

**Conclusion:**

Prevalence of repeated high SBP, DBP, and SDBP were within the expected distribution at the population level. Nonetheless, gestational diabetes mellitus, obesity, family history of hypertension, and prematurity increased the risk of repeated high blood pressure measured at two occasions 5 years apart.

## Background

Cardiovascular diseases are the leading causes of death in the world, and high blood pressure (BP) is among the cardiovascular diseases that most affect the adult population [1, 2]. Considered a silent disease, the clinical manifestation of arterial hypertension generally emerges in middle age as complications in target-organs such as kidney, heart, and brain. Adult hypertension precursors, however, are thought to originate in early life. Many perinatal and early life characteristics have been associated with the development of adult hypertension [3]. Longitudinal studies have shown that BP tracks through childhood and BP paths are set in the first years of life [4–7].

While the diagnosis of hypertension in adults is based on empirically determined cutoff values for systolic and diastolic BP, the diagnosis of hypertension in childhood is defined as the mean systolic and diastolic BP that is ≥95th percentile by age, sex and height on three or more occasions [8]. The European Society of Hypertension estimates the hypertension prevalence should be around 5.0% for children and adolescents up to 16 years of age, and it seems to have increased in the past decades due to the increased prevalence of obesity [9]. In a comprehensive systematic review of studies published up to 2013 carried out to assess the prevalence of high BP among adolescents, the pooled prevalence of high BP was 11.2% (13.0% for boys and 9.6% for girls) [10]. Thus, the ability to predict adult hypertension by measuring BP levels in childhood is of clinical and public health importance [11].

This study was planned to measure the prevalence of repeated (at 6 and 11 years of age) high systolic BP (SBP), high diastolic BP (DBP) and high systolic and diastolic BP (SDBP), and to investigate the association of maternal, perinatal and early life characteristics with repeated high SBP, DBP and SDBP, among participants of the Pelotas (Brazil) 2004 Birth Cohort.

## Methods

### Research setting and study design

During 2004, all the 4261 live births to mothers living in the urban area of the city of Pelotas (Brazil) were eligible to *The Pelotas 2004 Birth Cohort Study.* Of those, 4231 (99.3%) mothers consented to their children to being enrolled in the study. Trained fieldworkers in the five local maternity hospitals interviewed all mothers within the first 24 h after the delivery. Detailed information about socioeconomic, prenatal care, behavior, morbidity, and other maternal and newborn characteristics were gathered. Children in the cohort were followed-up at mean (standard deviation) ages of 3.0 (0.1), 11.9 (0.2), 23.9 (0.4), 49.5 (1.7), and 82.2 (4.0) months and at 6.7 (0.2) and 10.3 (0.5) years of life. A detailed description of the methodology of the cohort was given in previous publications [12–14]. The questionnaires and interviewer guides from all follow-up visits are available in electronic formats at [http://www.epidemio-ufpel.org.br/site/content/coorte_2004/index.php].

Data from the hospital interview and the 6- and 11-year follow-ups were used in this study. A total of 95 children died from birth to 6 years and three more between 6 and 11 years old. Response rates at 6- and 11-year follow-ups were 90.2 and 86.6%, respectively. The current analyses included 3182 children (75.2% of the original cohort) with full information on BP measures at 6 and 11 years. The follow-up visits were held at a clinic installed at the headquarters of the Federal University of Pelotas Epidemiologic Research Center. Several child characteristics were investigated, including detailed anthropometric and health conditions evaluation [13]. Trained interviewers applied standard and pre-codified questionnaires in the hospital and follow-up interviews.

### Outcome

A digital automatic OMRON sphygmomanometer© (model HEM 742) was used to measure BP in both follow-ups. BP was measured twice (2 min apart), on the children’s left arm, after remaining seated quietly for at least 5 min. Appropriate-sized cuffs were used for each child arm circumference (≤ 23 cm or > 23 cm). The mean systolic and diastolic BP values of the two measures were calculated and transformed into Z-scores by sex, age (in complete years) and height, following international recommendations [15, 16]. The outcome (repeated high SBP, DBP and SDBP) were defined, respectively, as systolic and diastolic BP Z-scores on the ≥95th percentile at the two visits (at 6- and 11-year follow-ups).

### Independent variables

Household monthly income and maternal and child characteristics obtained at the hospital interview were among the independent variables used. Family income in Brazilian Real at the month before the child’s birth was categorized into quintiles (the first quintile representing the poorest and the fifth quintile representing the wealthiest families). Medical diagnoses of maternal gestational diabetes mellitus and hypertension during pregnancy were reported by the mother (yes or no). Tobacco use during pregnancy (yes, no) as reported by the mother was defined as at least one daily cigarette during any trimester of the pregnancy. Maternal age was categorized as ≤20, 21–25, 26–30, and > 30 years.

The newborn birth weight was measured and recorded by the hospital staff with pediatric scales that were checked weekly by the research team using standard weights. Infants were categorized into low birth weight (< 2500 g) or normal weight (≥ 2500 g). Gestational age was calculated using the first day of the last normal menstrual period or estimated by obstetric ultrasound obtained before 20 gestational weeks when information about the last normal menstrual period was unreliable or not available. In the absence of both menstrual and ultrasound information, gestational age was estimated from the physical and neurological assessment of the newborn, employing the Dubowitz method [17]. Gestational age was categorized into < 34 weeks, 34–36 weeks, and ≥ 37 weeks. Weight-for-gestational age variable was generated as per the standard population curve proposed by Williams et al. [18] and classified as small (birth weight lower than the 10th centile), adequate (birthweight between the 10th and the 90th centile) or large for gestational age (birth weight above the 90th centile) for a specific completed gestational age and sex.

The child’s skin color (reported by the mother) and family history of hypertension were collected at the 6-year follow-up visit. Skin color was later categorized into white and non-white. Information on family history of hypertension in the parents’ first-degree relatives (grand-parents of the child) was categorized as none, from mother, father, or both.

Information on the child physical activity pattern, BMI-for-age, daily salt intake, and weekly consumption of package chips was obtained at the 11-year follow-up. Children were considered as active if they performed structured physical activities (with adult/teacher/coach intervention) in private services after school period. The child weight was measured with a high precision scale (0.01 kg) that was part of the BODPOD® machine (Cosmed, Italy, http://goo.gl/7jzfLc). Height was measured by trained anthropometrists using a Harpenden metal stadiometer, with 1 mm precision (Holtain, Crymych, UK). Children were classified by BMI z-score into “normal weight” (< + 1 SD), “overweight” (≥ + 1 to < + 2 SD) and “obese” (≥ + 2 SD) [19]. A validated semi-quantitative food frequency questionnaire (FFQ) was administered to the mother to estimate the child mean daily sodium intake. The children assisted with the FFQ report by answering questions on frequency and portion sizes of a list containing 88 different foods. The recall period was the past 12 months. We reconfigured the 12-month-food consumption to daily consumption, with all portions standardized at 100 g. The sodium content of each food was estimated based on The Brazilian Food Composition Table [20]. Daily sodium intake in milligrams was then calculated, which was later categorized in quintiles (the first quintile representing the lowest sodium intake and the fifth quintile the highest). Information on weekly consumption of package chips was investigated and classified in none, up to 1, 2, and ≥ 3.

### Statistical analyses

Firstly, the maternal and child characteristics of the sample located at the 11-year follow-up and of those included in the analyses were compared to the whole cohort. Then, we calculated the prevalence (95% CI) of repeated high SBP, DBP, and SDBP with a 95% confidence interval, by the independent variables. The associations were assessed by Fisher’s exact chi-square test.

Crude and adjusted logistic regression [21] was used to obtain odds ratios with 95% CI for the outcome. The backward selection strategy was employed in the multivariable analyses. The selection started with all variables in the model, then the variables with the largest *p*-value were removed one-by-one, thus stopping when all remaining variables were associated with the outcome at a *p*-value ≤0.20. All analyses were performed with Stata software version 12.0 with a statistical significance level of *p*-value < 0.05.

The Medical Ethics Committee of Faculty of Medicine of the Federal University of Pelotas, affiliated with the Brazilian National Commission for Research Ethics (CONEP), approved the study protocol of all follow-ups of the Pelotas 2004 Birth Cohort. At each stage of the study, the mothers or legal guardians gave written informed consent. At 11 years of age, written informed consent was also obtained from the cohort members.

## Results

Table 1 describes the characteristics of mothers and children enrolled in the Pelotas 2004 Birth Cohort, the percentage located at the 11-year follow-up, and the proportion included in the current analyses. There was a significant difference in the follow-up rate for family income, mothers’ age at childbirth, and gestational diabetes mellitus. Losses of follow-up and missing information in the outcome were higher among children from the most impoverished families and born to mothers aged 21–25 years and without gestational diabetes mellitus.

**Table 1:** Characteristics of mothers and children enrolled in the Pelotas 2004 Birth Cohort

Characteristics (%)	Original cohort (*N* = 4231)	Percentage located at 11 years (*N* = 3565)	*p-value* ^*a*^	Analyzed sample (*N* = 3182)	*p-value* ^*b*^
Maternal Characteristics					
Household income (quintiles)			*< 0.001*		*< 0.001*
1 (poorest)	871 (20.6)	82.7		*80.6*	
2	854 (20.2)	86.3		*85.1*	
3	816 (19.3)	88.7		*87.6*	
4	858 (20.3)	89.6		*88.7*	
5 (wealthiest)	830 (19.6)	84.3		*82.4*	
Age at childbirth (years)			*0.001*		*0.001*
≤ 20	1032 (24.4)	85.2		*83.8*	
21–25	1129 (26.7)	83.8		*82.1*	
26–30	924 (21.9)	86.6		*85.2*	
31+	1142 (27.0)	89.4		*88.2*	
Smoking during pregnancy			*0.059*		*0.079*
No	3067 (72.5)	86.9		*85.5*	
Yes	1162 (27.5)	84.6		*83.2*	
Gestational diabetes mellitus			*0.011*		*0.008*
No	4100 (97.0)	86.0		84.6	
Yes	126 (3.0)	94.2		93.7	
Hypertension^c^ in pregnancy			*0.623*		*0.546*
No	3220 (76.3)	86.2		84.7	
Yes	1001 (23.7)	86.8		85.6	
Child Characteristics					
Sex			*0.498*		*0.471*
Male	2195 (51.9)	85.9		84.5	
Female	2034 (48.1)	86.6		85.3	
Birth weight			0.776		*0.517*
≥ 2500 g	3803 (90.0)	86.3		85.0	
< 2500 g	423 (10.0)	85.8		83.6	
Gestational age (weeks)			*0.202*		*0.171*
< 34	140 (3.3)	82.1		*79.1*	
34–36	472 (11.2)	88.3		*86.8*	
≥ 37	3603 (85.5)	86.2		84.8	
Weight-for-gestational age			*0.628*		*0.585*
Small	526 (12.5)	85.7		84.3	
Adequate	3390 (80.4)	86.2		84.8	
Large	300 (7.1)	88.1		87.0	
Skin color			*0.159*		*0.095*
White	2726 (68.2)	88.3		87.1	
Non-white	1272 (31.8)	89.8		89.0	

^a^Chi-square test to compare children enrolled in the 2004 Pelotas Birth Cohort and percentage located at the 11-year follow-up

^b^Chi-square test to compare children enrolled in the 2004 Pelotas Birth Cohort and proportion included in the analyses

^c^*HBP* high blood pressure

The description of the analyzed sample and prevalence (95% CI) of repeated high SBP, DBP, and SDBP, by maternal and child characteristics are shown in Table 2. Most of the analyzed children (67.1%) were white. Medical diagnosis of gestational diabetes mellitus and hypertension during pregnancy were reported by 3.3 and 24.0% of the mothers, respectively. Maternal age at childbirth was 31 years or more for 28.1% of the mothers. Family history of hypertension was positive for more than one-quarter of the sample (26.2%), and 26.9% of the mothers smoked during pregnancy. Prevalence of low birth weight was 8.5%, preterm births accounted for 13.4% of the sample, and about 12.3% of the children were small for gestational age at birth (Table 2).

**Table 2:** Description of the analyzed sample and prevalence (95% confidence interval) of repeated high blood pressure

Characteristics	*N* (%)	HSBP^a^ (*N* = 53)	HDBP^b^ (*N* = 74)	HSDBP^c^ (*N* = 40)
Sex		*0.018*	*0.019*	*0.525*
Male	1637 (51.45)	2.2 (1.5–2.9)	1.7 (1.1–2.3)	1.4 (0.8–2.0)
Female	1545 (48.55)	1.1 (0.6–1.6)	3.0 (2.1–3.8)	1.1 (0.6–1.6)
Skin color		*0.377*	*0.260*	*0.235*
White	2136 (67.13)	1.8 (1.3–2.4)	2.1 (1.5–2.7)	1.1 (0.6–1.5)
Non-white	1046 (32.87)	1.3 (0.6–2.0)	2.8 (1.8–3.8)	1.6 (0.9–2.4)
Gestational diabetes		*0.029*	*0.735*	*0.144*
No	3074 (96.70)	1.6 (1.1–2.0)	2.3 (1.8–2.8)	1.2 (0.8–1.6)
Yes	105 (3.30)	4.8 (0.7–8.9)	2.9 (−0.3–6.1)	2.9 (−0.3–6.1)
Hypertension in pregnancy		*0.143*	*0.409*	*0.580*
No	2413 (75,95)	1.5 (1.0–1.9)	2.2 (1.6–2.8)	1.2 (0.8–1.6)
Yes	764 (24,05)	2.2 (1.2–3.3)	2.7 (1.6–3.9)	1.4 (0.6–2.3)
Mothers’ age at childbirth		*0.832*	*0.081*	*0.181*
≤ 20	768 (24.15)	1.6 (0.7–2.4)	1.3 (0.5–2.1)	0.8 (0.2–1.4)
21–25	822 (25.85)	1.5 (0.6–2.3)	2.4 (1.4–3.5)	1.0 (0.3–1.6)
26–30	697 (21.92)	1.6 (0.7–2.5)	3.3 (2.0–4.6)	2.0 (1.0–3.1)
> 30	893 (28.08)	2.0 (1.1–2.9)	2.4 (1.4–3.3)	1.3 (0.6–2.1)
Family history hypertension		*0.442*	*0.021*	*0.489*
None	2079 (73.80)	1.6 (1.1–2.2)	2.2 (1.6–2.8)	1.2 (0.7–1.7)
From mother	354 (12.57)	1.7 (0.3–3.0)	1.7 (0.3–3.0)	1.1 (0.0–2.2)
From father	316 (11.22)	2.5 (0.8–4.3)	2.5 (0.8–4.3)	1.3 (0.0–2.5)
From both	68 (2.41)	2.9 (−1.1–7.0)	8.8 (2.0–15.6)	2.9 (−1.1–7.0)
Household income (quintiles)		*0.148*	*0.053*	*0.256*
1 (poorest)	608 (19.11)	1.5 (0.5–2.4)	3.6 (2.1–5.1)	1.3 (0.4–2.2)
2	649 (20.40)	0.9 (0.2–1.7)	1.7 (0.7–2.7)	1.1 (0.3–1.9)
3	634 (19.92)	2.1 (0.9–3.2)	1.3 (0.4–2.1)	1.1 (0.3–1.9)
4	687 (21.59)	1.3 (0.5–2.2)	2.3 (1.2–3.5)	0.7 (0.1–1.4)
5 (wealthiest)	604 (18.98)	2.6 (1.4–3.9)	2.8 (1.5–4.1)	2.2 (1.0–3.3)
Smoking during pregnancy		*0.463*	*0.144*	*0.935*
No	2325 (73.07)	1.6 (1.1–2.2)	2.6 (1.9–3.2)	1.2 (0.8–1.7)
Yes	857 (26.93)	1.8 (0.9–2.6)	1.6 (0.8–2.5)	1.3 (0.5–2.0)
Birth weight		*0.452*	*0.057*	*1.000*
≥ 2500 g	2911 (91.48)	1.6 (1.2–2.1)	2.2 (1.6–2.7)	1.2 (0.8–1.6)
< 2500 g	271 (8.52)	2.2 (0.5–4.0)	4.1 (1.7–6.4)	1.5 (0.0–2.9)
Weight-for-gestational age		*0.752*	*0.715*	*0.772*
Small	392 (12.33)	2.0 (0.6–3.4)	2.0 (0.6–3.4)	2.0 (0.6–3.4)
Adequate	2554 (80.31)	1.6 (1.1–2.1)	2.3 (1.7–2.9)	1.2 (0.8–1.6)
Large	234 (7.36)	1.7 (0.0–3.4)	3.0 (0.8–5.2)	0.9 (0.3–0.2)
Gestational age (weeks)		*0.178*	*0.011*	*0.300*
< 34	72 (2.26)	1.4 (−1.3–4.1)	8.3 (1.9–14.8)	1.4 (− 1.3–4.1)
34–36	355 (11.16)	2.8 (1.1–4.5)	2.5 (0.9–4.2)	2.3 (0.7–3.8)
≥ 37	2753 (86.57)	1.5 (1.1–2.0)	2.1 (1.6–2.7)	1.1 (0.7–1.5)
Physical activity		*0.441*	*0.896*	*0.138*
Active	896 (28.40)	1.3 (0.6–2.1)	2.2 (1.3–3.2)	1.1 (0.4–1.8)
No active	2259 (71.60)	1.8 (1.2–2.3)	2.4 (1.8–3.0)	1.2 (0.8–1.7)
Sodium intake (mg) (quintiles)		*0.428*	*0.769*	*0.858*
1 (lowest)	622 (19.60)	1.0 (0.2–1.7)	2.1 (1.0–3.2)	1.4 (0.5–2.4)
2	644 (20.29)	1.7 (0.7–2.7)	3.0 (1.6–4.3)	1.4 (0.5–2.3)
3	640 (20.16)	2.2 (1.1–3.3)	2.5 (1.3–3.7)	1.6 (0.6–2.5)
4	632 (19.91)	1.4 (0.5–2.3)	1.9 (0.8–3.0)	0.8 (0.1–1.5)
5 (highest)	636 (20.04)	2.0 (0.9–3.1)	2.2 (1.1–3.3)	1.1 (0.3–1.9)
Weekly consumption of package chips		*0.229*	*0.748*	*0.721*
None	299 (9.42)	2.3 (0.6–4.1)	2.3 (0.6–4.1)	2.3 (0.6–4.1)
Up to 1 package	1586 (49.97)	1.9 (1.2–2.6)	2.1 (1.4–2.8)	1.3 (0.8–1.9)
2 packages	758 (23.88)	0.9 (0.2–1.6)	2.8 (1.6–3.9)	0.8 (0.2–1.4)
≥ 3 packages	531 (16.73)	1.7 (0.6–2.8)	2.4 (1.1–3.8)	1.1 (0.2–2.0)
*BMI-for-age Z score (11 years old)*		*0.011*	*1.000*	*0.240*
Normal	1769 (55.59)	1.1 (0.6–1.6)	2.4 (1.7–3.1)	1.1 (0.6–1.6)
Overweight	712 (22.38)	1.8 (0.8–2.8)	2.2 (1.2–3.3)	1.8 (0.8–2.8)
Obesity	701 (22.03)	2.9 (1.6–4.1)	2.3 (1.2–3.4)	2.9 (1.6–4.1)
Total		1.7 (1.2–2.1)	2.3 (1.8–2.9)	1.2 (0.9–1.6)

^*^All *P*-values calculated by Fisher’s Exact chi-square test

^a^*HSBP* High systolic blood pressure, ^b^*HDBP* High diastolic blood pressure, ^c^*HSDBP* High systolic and diastolic blood pressure

At 11 years of age, 28.4% of the children were physically active. Median daily salt intake (interquartile interval) was 2296.3 mg (1663.1 – 3277.1 mg), and about 17% of the children usually consumed ≥3 package chips per week. Prevalence of excessive weight at 11 years was 44.4% (22.4% were overweight, and 22.0% were obese) (Table 2).

Systolic and diastolic BP in the whole sample had a normal distribution. Mean and standard deviation (SD) of systolic and diastolic BP at 6 years were 99.1 (9.1) mmHg and 60.5 (8.8) mmHg, respectively, and 113.1 (11.3) mmHg and 66.1 (8.7) mmHg at 11 years, respectively. Systolic BP among children with repeated high SBP had a normal distribution, with mean values of 122.3 (4.4) mmHg at 6 years and 120.7 (12.7) mmHg at 11. Diastolic BP among children with repeated high DBP at 6 and 11 years had an asymmetric distribution, with median (IQR) values for DBP of 82.5 (80.0–85) mmHg at 6 years and 69.0 (62.5–75.0) mmHg at 11. Within the group of children with repeated high SDBP, the systolic BP distribution was normal, and diastolic BP was asymmetric. The mean values of systolic BP within this group were 125.8 (7.4) mmHg at 6 years and 117.5 (12.9) mmHg at 11, whereas the median (IQR) values of diastolic BP were 85.5 (82.5–92.3) mmHg at 6 years and 69.3 (63.5–76.8) mmHg at 11.

**Fig. 1:**
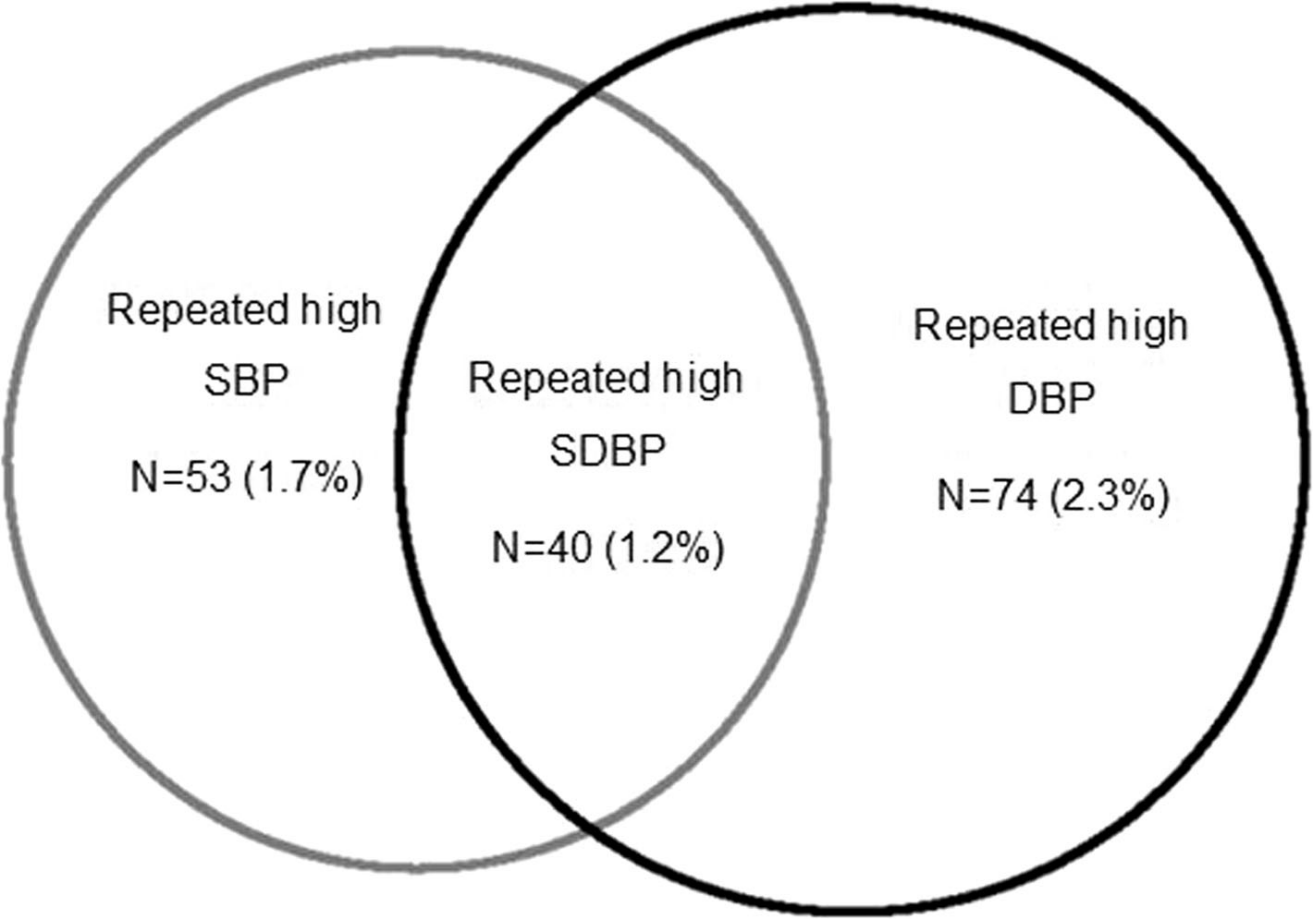
Prevalence of repeated high systolic (SBP), diastolic (DBP) and both blood pressure (SDBP)

In Fig. 1, the prevalence of repeated high SBP was 1.7% (1.2–2.1%), of which 2.2% (1.5–2.9%) in boys and 1.1% (0.6–1.6%) in girls. The prevalence of repeated high DBP and repeated high SDBP was 2.3% (1.8–2.9%) and 1.2% (0.9–1.6%), respectively (Fig. 1).

Table 3 presents crude and adjusted OR with 95% CI for repeated high SBP. In adjusted analyses, repeated high SBP was associated with males, gestational diabetes mellitus, and BMI-for-age Z score at 11 years of age. The likelihood of girls evidencing repeated high SBP was 49% lower than boys (OR = 0.51; 0.29–0.92). Offspring of mothers who reported a medical diagnosis of gestational diabetes mellitus had an almost threefold higher risk of repeated high SBP (OR = 2.92; 1.13–7.58) when compared to those from mothers without gestational diabetes. Repeated high SBP among obese children was 2.44 (1.29–4.59) times more likely than among those with normal BMI-for-age (Table 3).

**Table 3:** Analyses for repeated high systolic blood pressure by maternal and child characteristics

Characteristics	Repeated High Systolic Blood Pressure					
	Crude OR	(95% CI)	*P* ^*^	Adjusted OR	(95% CI)	*P* ^*^
Sex			*0.015*			*0.025*
Male	1.00	–		1.00	–	
Female	0.49	(0.28–0.88)		0.51	(0.29–0.92)	
Skin color			*0.304*			*0.789*
White	1.00	–		1.00	–	
Non-white	0.73	(0.39–1.35)		0.91	(0.47–1.76)	
Gestational diabetes			*0.038*			*0.027*
No	1.00	–		1.00	–	
Yes	3.15	(1.23–8.09)		2.92	(1.13–7.58)	
Hypertension in pregnancy			*0.155*			*0.236*
No	1.00	–		1.00	–	
Yes	1.55	(0.86–2.78)		1.44	(0.79–2.64)	
Maternal age at childbirth			*0.818*			*0.866*
≤ 20	1.00	–		1.00	–	
21–25	0.93	(0.42–2.09)		0.88	(0.38–2.04)	
26–30	1.01	(0.44–2.30)		0.68	(0.67–3.41)	
> 30	1.30	(0.62–2.71)		0.89	(0.30–6.25)	
Family history hypertension			*0.657*			*0.704*
None	1.00	–		1.00	–	
From mother	1.04	(0.43–2.49)		0.80	(0.31–2.06)	
From father	1.56	(0.72–3.41)		1.48	(0.66–3.31)	
From both	1.82	(0.43–7.75)		1.32	(0.29–6.00)	
Household income (quintiles)			*0.144*			*0.240*
1 (poorest)	0.55	(0.24–1.26)		0.59	(0.25–1.37)	
2	0.34	(0.13–0.88)		0.37	(0.14–0.97)	
3	0.77	(0.37–1.61)		0.79	(0.37–1.70)	
4	0.49	(0.21–1.11)		0.51	(0.22–1.17)	
5 (wealthiest)	1.00	–		1.00	–	
Smoking during pregnancy			*0.822*			*0.885*
No	1.00	–		1.00	–	
Yes	1.07	(0.59–1.96)		1.05	(0.54–2.04)	
Birth weight			*0.480*			*0.501*
≥ 2500 g	1.00	–		1.00	–	
< 2500 g	1.38	(0.58–3.26)		1.44	(0.50–4.12)	
Weight-for-gestational age			*0.830*			*0.972*
Small	1.28	(0.59–2.74)		0.93	(0.33–2.64)	
Adequate	1.00	–		1.00	–	
Large	1.07	(0.38–3.00)		0.89	(0.30–2.64)	
Gestational age (weeks)			*0.252*			*0.135*
< 34	0.91	(0.12–6.70)		1.05	(0.14–7.79)	
34–36	1.87	(0.93–3.76)		2.05	(1.01–4.16)	
≥ 37	1.00	–		1.00	–	
Physical activity at 11 years			*0.381*			*0.290*
Active	1.00	–		1.00	–	
No active	1.33	(0.69–2.54)		1.43	(0.74–2.76)	
Sodium intake (mg) (quintiles)			*0.416*			*0.322*
1 (lowest)	1.00	–		1.00	–	
2	1.78	(0.66–4.85)		1.80	(0.64–5.05)	
3	2.30	(0.88–6.01)		2.63	(0.97–7.14)	
4	1.48	(0.52–4.19)		1.90	(0.65–5.60)	
5 (highest)	2.14	(0.81–5.67)		2.82	(0.97–8.19)	
Weekly consumption of chips			*0.236*			*0.387*
None	1.00	–		1.00	–	
Up to 1 package	0.80	(0.35–1.85)		1.02	(0.41–2.53)	
2 packages	0.39	(0.14–1.12)		0.51	(0.17–1.58)	
≥ 3 packages	0.72	(0.27–1.95)		1.15	(0.39–3.39)	
*BMI-for-age Z score (11 years old)*			*0.014*			*0.022*
Normal	1.00	–		1.00	–	
Overweight	1.63	(0.80–3.29)		1.64	(0.81–3.31)	
Obesity	2.57	(1.37–4.80)		2.44	(1.29–4.59)	

^*^P-values were calculated by Wald Test to heterogeneity

Crude and adjusted analyses for repeated high DBP are shown in Table 4. In the adjusted analyses, repeated high DBP was associated with female sex, grandparents’ history of hypertension, and prematurity. The likelihood of girls having repeated high DBP was 67% higher than boys (OR = 1.67; 1.01–2.76). Grandparents’ history of hypertension from both sides increased 3.95 times (95%CI 1.59–9.85) the likelihood of repeated high DBP, when compared to children without a family history of hypertension; and preterm children at < 34 weeks were 4.15 (1.73–9.95) times more likely to show repeated high DBP than those born at term (Table 4).

**Table 4:** Analyses for repeated high diastolic blood pressure according to maternal and child characteristics

Characteristics	Repeated High Diastolic Blood Pressure					
	Crude OR	(95% CI)	P*	Adjusted OR	(95% CI)	P*
Sex			*0.017*			*0.047*
Male	1.00	–		1.00	–	
Female	1.76	(1.10–2.84)		1.67	(1.01–2.76)	
Skin color			*0.249*			*0.182*
White	1.00	–		1.00	–	
Non-white	1.33	(0.83–2.13)		1.43	(0.84–2.43)	
Gestational diabetes			*0.723*			*0.928*
No	1.00	–		1.00	–	
Yes	1.24	(0.39–4.02)		1.06	(0.31–3.57)	
Hypertension in pregnancy			*0.387*			*0.520*
No	1.00	–		1.00	–	
Yes	1.26	(0.75–2.10)		1.22	(0.67–2.22)	
Maternal age at childbirth			*0.078*			*0.114*
≤ 20	1.00	–		1.00	–	
21–25	1.89	(0.88–4.06)		2.42	(1.04–5.66)	
26–30	2.59	(1.22–5.47)		2.76	(1.18–6.48)	
> 30	1.83	(0.85–3.90)		1.98	(0.83–4.70)	
Family history hypertension			*0.040*			*0.018*
None	1.00	–		1.00	–	
From mother	0.76	(0.32–1.80)		0.69	(0.29–1.65)	
From father	1.15	(0.54–2.46)		1.12	(0.52–2.41)	
From both	4.28	(1.76–10.39)		3.95	(1.59–9.85)	
Household income (quintiles)			*0.051*			*0.062*
1 (poorest)	1.30	(0.68–2.47)		1.37	(0.67–2.83)	
2	0.60	(0.28–1.28)		0.60	(0.25–1.40)	
3	0.44	(0.19–1.03)		0.41	(0.17–1.02)	
4	0.82	(0.41–1.64)		0.79	(0.39–1.61)	
5 (wealthiest)	1.00	–		1.00	–	
Smoking during pregnancy			*0.103*			*0.089*
No	1.00	–		1.00	–	
Yes	0.63	(0.35–1.13)		0.56	(0.28–1.09)	
Birth weight			*0.070*			*0.836*
≥ 2500 g	1.00	–		1.00	–	
< 2500 g	1.91	(1.00–3.67)		1.11	(0.40–3.09)	
Weight-for-gestational age			*0.753*			*0.783*
Small	0.88	(0.42–1.86)		0.90	(0.36–2.24)	
Adequate	1.00	–		1.00	–	
Large	1.30	(0.59–2.89)		1.32	(0.57–3.02)	
Gestational age (weeks)			*0.025*			*0.020*
< 34	4.15	(1.73–9.95)		4.08	(1.52–10.96)	
34–36	1.19	(0.58–2.42)		1.00	(0.45–2.23)	
≥ 37	1.00	–		1.00	–	
Physical activity at 11 years of age			*0.790*			*0.695*
Active	1.00			1.00	–	
Inactive	1.07	(0.64–1.80)		1.12	(0.63–1.99)	
Sodium intake (mg) (quintiles)			*0.764*			*0.523*
1 (lowest)	1.00	–		1.00	–	
2	1.42	(0.70–2.91)		1.68	(0.79–3.56)	
3	1.20	(0.57–2.52)		1.07	(0.48–2.39)	
4	0.91	(0.41–2.00)		0.92	(0.39–2.20)	
5 (highest)	1.05	(0.49–2.26)		1.15	(0.50–2.68)	
Weekly consumption of package chips			*0.779*			*0.454*
None	1.00	–		1.00	–	
Up to 1 package	0.89	(0.39–2.02)		0.77	(0.33–1.82)	
2 packages	1.19	(0.50–2.83)		1.22	(0.49–3.05)	
≥ 3 packages	1.05	(0.41–2.65)		1.27	(0.44–3.67)	
*BMI-for-age (11 years old – z score)*			*0.978*			*0.946*
Normal	1.00	–		1.00	–	
Overweight	0.95	(0.53–1.69)		1.02	(0.55–1.88)	
Obesity	0.96	(0.54–1.72)		0.91	(0.47–1.75)	

* *P*-values were calculated by Wald Test to heterogeneity

*HBP* High Blood Pressure

As shown in Table 5, repeated high SDBP at the two occasions was not associated with any of the independent variables investigated in crude or the adjusted analysis.

**Table 5:** Analyses for repeated high systolic and diastolic blood pressure according to maternal and child characteristics

Characteristics	Repeated High Systolic and Diastolic Blood Pressure					
	Crude OR	(95% CI)	*P* ^*^	Adjusted OR	(95% CI)	*P* ^*^
Sex			*0.440*			*0.790*
Male	1.00	–		1.00	–	
Female	0.78	(0.42–1.47)		0.91	(0.45–1.84)	
Skin color			*0.201*			*0.180*
White	1.00	–		1.00	–	
Non-white	1.52	(0.81–2.85)		1.54	(0.82–2.91)	
Gestational diabetes			*0.197*			*0.198*
No	1.00	–		1.00	–	
Yes	2.41	(0.73–7.96)		2.20	(0.66–7.31)	
HBP in pregnancy			*0.613*			*0.686*
No	1.00	–		1.00	–	
Yes	1.20	(0.60–2.42)		1.16	(0.56–2.39)	
Mothers’ age at childbirth			*0.177*			*0.159*
≤ 20	1.00	–		1.00	–	
21–25	1.25	(0.43–3.61)		1.28	(0.44–3.73)	
26–30	2.60	(0.99–6.81)		2.71	(1.03–7.13)	
> 30	1.73	(0.65–4.63)		1.79	(0.67–4.83)	
Family history hypertension			*0.748*			*0.703*
None	1.00	–		1.00	–	
From mother	0.94	(0.32–2.71)		0.83	(0.27–2.61)	
From father	1.05	(0.36–3.05)		1.01	(0.34–3.01)	
From both	2.49	(0.58–10.73)		2.36	(0.49–11.35)	
Household income (quintiles)			*0.252*			*0.352*
1 (poorest)	0.61	(0.25–1.47)		0.63	(0.24–1.63)	
2	0.50	(0.20–1.25)		0.50	(019–1.32)	
3	0.51	(0.20–1.28)		0.56	(0.21–1.46)	
4	0.33	(0.12–0.94)		0.36	(0.13–1.03)	
5 (wealthiest)	1.00	–		1.00		
Smoking during pregnancy			*0.935*			*0.939*
No	1.00	–		1.00		
Yes	1.03	(0.51–2.07)		1.03	(0.44–2.41)	
Birth weight			*0.741*			*0.319*
≥ 2500 g	1.00	–		1.00	–	
< 2500 g	1.20	(0.42–3.39)		0.51	(0.13–1.92)	
Weight-for-gestational age			*0.346*			*0.355*
Small	1.75	(0.80–3.85)		1.67	(0.76–3.69)	
Adequate	1.00	–		1.00	–	
Large	0.73	(0.17–3.05)		0.68	(0.16–2.89)	
Gestational age (weeks)			*0.259*			*0.186*
< 34	1.24	(0.17–9.19)		1.28	(0.17–9.56)	
34–36	2.02	(0.92–4.44)		2.09	(0.95–4.62)	
≥ 37	1.00	–		1.00	–	
Physical activity at 11 years of age			*0.773*			*0.380*
Active	1.00	–		1.00	–	
No Active	1.11	(0.54–2.30)		1.40	(0.66–2.96)	
Sodium intake (mg) (quintiles)			*0.713*			*0.842*
1 (lowest)	1.00	–		1.00	–	
2	0.97	(0.38–2.45)		1.15	(0.45–2.96)	
3	1.08	(0.44–2.68)		1.12	(0.43–2.93)	
4	0.54	(0.18–1.63)		0.58	(017–1.98)	
5 (highest)	0.76	(0.28–2.05)		0.95	(0.30–3.01)	
Weekly consumption of chips			*0.271*			*0.249*
None	1.00	–		1.00	–	
Up to 1 package	0.56	(0.24–1.33)		0.57	(0.24–1.38)	
2 packages	0.33	(0.11–1.00)		0.32	(0.11–0.99)	
≥ 3 packages	0.48	(0.16–1.43)		0.47	(0.15–1.44)	
*BMI-for-age (11 years old – Z score)*			*0.567*			*0.838*
Normal	1.00	–		1.00	–	
Overweight	1.31	(0.61–2.84)		1.15	(0.48–2.77)	
Obesity	1.47	(0.70–3.10)		1.29	(0.55–3.06)	

*P-values were calculated by Wald Test to heterogeneity

## Discussion

This study showed that the prevalence of repeated high SBP (1.7%), DBP (2.3%) and SDBP (1.2%), as measured on two occasions 5 years apart (6 and 11 years of age), were within the normal expected distribution for population-based studies. Nonetheless, the prevalence of repeated high SBP among boys (almost 50% higher than in girls), in children from mothers that reported gestational diabetes mellitus (4.8%) and in those with obesity was above the expected distribution. In the same way, the prevalence of repeated high DBP among girls (3.0%), in those born before 34 weeks of gestation (8.3%) and from families with a history of hypertension from both sides (8.8%) exceeded the expected prevalence for a normal distribution.

Our findings are in line with longitudinal studies that showed that children with elevated BP levels, even when within the normal limits, tend to show a progression along life, with higher levels than other individuals and greater probability of becoming an adult with hypertension [4, 5, 7]. Results from the 27-year tracking of cardiovascular risk factors at the Cardiovascular Risk in Young Finns Study, a multicenter study, showed robust tracking of BP between childhood and adulthood, and that high BP at any age in childhood (6 and 9 years) or adolescence (12, 15 and 18 years) in male and female participants, increased the likelihood to and evidenced a high accuracy of predicting abnormal adult BP (sensitivity and specificity rates about 62.4 and 63.8%, respectively, for both sexes at all ages) [4].

In the same way, at 23 years of age, participants of the Birth to Twenty Plus cohort in urban Soweto, South Africa, within the highest systolic and diastolic BP trajectories were at increased risk of elevated BP. Those in the highest systolic trajectory path had a fourfold increased risk, and those in the highest diastolic trajectory, a 5-fold increased risk, thus suggesting that risk for elevated BP in adulthood may be set in childhood and adolescence [7].

### Repeated high SBP

In our study, we found an association between repeated high SBP and male sex, maternal medical diagnosis of gestational diabetes mellitus, and obesity at 11 years. Association between males and higher BP has been described in several studies with children participants [22–25]. Although the mechanisms responsible for the sex differences in blood pressure control are not clear, there is significant evidence that androgens, such as testosterone, play an essential role in sex-associated differences in blood pressure regulation [26–28].

Exposure to gestational diabetes mellitus was associated with a three-fold increase in the risk of repeated high SBP. In a meta-analysis, Aceti et al. [29] reported higher SBP in the offspring of diabetic mothers, and the association was stronger in boys than in girls. The plausibility of this association is not entirely understood, but the hyperinsulinemia induced by a mother with gestational diabetes appears to increase the offspring’s arterial stiffness [30]. Furthermore, maternal gestational diabetes mellitus is associated with both maternal and offspring obesity, which are also associated with offspring hypertension [31].

Obesity and hypertension in young people have been increasing in the last decades [32], and a strong relationship between obesity and high BP among children has been reported in several studies [9, 33–35]. We found an association of obesity only with repeated high SBP. The mechanism that contributes to elevated SBP in obese individuals is a combination of factors that elevate systemic vascular resistance [36, 37], including the activation of the sympathetic nervous system [38].

### Repeated high DBP

Repeated high DBP in our study was associated with female sex, family history of hypertension, and preterm birth. Similar to our results, a case-control study with children under 5 years of age found that preterm children had higher systolic and diastolic BP than those born at term [39]. Additionally, results from previous studies investigating adult cardiometabolic risks have suggested a linear relationship between shorter gestation duration and higher BP in adults [40–42]. Preterm births have been associated with abnormalities on vascular growth [43], impaired autonomic reflexes [44], and kidney abnormalities [45]. Also, stress experienced in the perinatal period and hospitalization in neonatal intensive care units may affect the hyperactivity of the sympathetic-adrenal function and consequently increase the risk for HBP [46].

The association of repeated high DBP with a family history of hypertension highlights the importance of genetic factors in the genesis of high BP. Polymorphisms and the renin-angiotensin-aldosterone system seem to be affected by the hereditability [47].

### Strengths and limitations of the study

One of the strengths of this study is the longitudinal design that ensures the time sequence of predictors and outcomes. The large number of children followed-up from birth with a low rate of attrition and the available information on several predictors, collected using standardized methodology, are also among the strengths.

Among the limitations, while blood pressure measurements had been done following international recommendations, factors such as white coat syndrome and circadian cycle variation (measurements in different days and periods) may influence individual’s blood pressure [48]. Differential losses in the analyzed sample may have affected the prevalence of HBP. Also, daily sodium intake, weekly consumption of package chips, reporting a medical diagnosis of gestational diabetes mellitus and hypertension in pregnancy may have been influenced by information bias.

## Conclusions

Male sex, gestational diabetes mellitus, and obesity increased the risk of repeated high SBP, whereas female sex, family history of hypertension and prematurity increased the risk of repeated high DBP on two occasions 5 years apart. The screening and management of gestational diabetes mellitus and the prevention of preterm births are potential strategies of primary prevention of repeated high BP from childhood to early adolescence.

## Data Availability

The datasets used and analyzed during the current study are available from the corresponding author on reasonable request.

## Abbreviations

BP: Blood pressure
DBP: Diastolic blood pressure
HBP: High blood pressure
SBP: Systolic blood pressure
SDBP: Systolic and diastolic blood pressure
